# Analyzing the students’ mathematical creative thinking ability in terms of self-regulated learning: How do we find what we are looking for?

**DOI:** 10.1016/j.heliyon.2024.e24871

**Published:** 2024-01-20

**Authors:** Hayatun Nufus, Ramon Muhandaz, Hasanuddin, Erdawati Nurdin, Rezi Ariawan, Rira Jun Fineldi, Isnaria Rizki Hayati, Dominikus David Biondi Situmorang

**Affiliations:** a Department of Mathematics Education, Universitas Islam Negeri Sultan Syarif Kasim Riau, Indonesia; b Department of Mathematics Education, Islamic University of Riau, Indonesia; c 1 Junior High School in Kampar, Indonesia; d Department of Guidance and Counseling, Universitas Riau, Pekanbaru, Riau, Indonesia; e Department Guidance and Counseling, Faculty of Education and Language, Atma Jaya Catholic University of Indonesia, Indonesia

**Keywords:** Profile, Self-regulated learning, Students' mathematical creative thinking ability

## Abstract

This study aims to analyze the mathematical creative thinking ability of grade VIII students at a State Junior High School in Riau, Indonesia, in terms of self-regulated learning. A qualitative design was employed, utilizing descriptive methods through a case study approach, and the participants included 28 students of grade VIII-C. To ensure data accuracy, a data triangulation technique was employed, which included a test for mathematical creative thinking skills, self-regulated learning questionnaires, introductory questionnaires, and interview guidelines. The validity of the data was measured using the Miles and Huberman technique, which involved data reduction, data presentation, and drawing conclusions. The results showed that the mathematical creative thinking skills of students were relatively low. Furthermore, it was discovered that self-regulated learning was significantly correlated with creative thinking ability. Specifically, students who exhibited high levels of self-regulated learning demonstrated higher creative thinking abilities than their peers with moderate or low levels. Similarly, participants with moderate self-regulated learning displayed sufficient abilities, while those with low self-regulated learning exhibited poor creative thinking abilities.

## 1 Introduction

Education is very crucial in the development of the globalization era, which presents numerous differences, distances, and challenges. To address these differences and challenges effectively, it is essential to provide education that enhances students’ awareness and understanding of the tendencies, fears, and threats associated with globalization. In particular, schools have a significant responsibility in ensuring that students are equipped with the knowledge and skills needed to address these issues [1].

In a constantly changing and uncertain world, schools have become one of the most stable institutions that promote positive values. Based on the perspective of numerous individuals, schools play a significant role in addressing complex situations, hence the need to develop a well-defined curriculum. Moreover, this institution needs to educate students on the fact that learning is a collaborative process requiring participation from both teachers and students. This approach can help instill a sense of responsibility in students, enabling them to take ownership of their learning and make the most of their educational opportunities [1].

Mathematics, as a subject of education, can be characterized in many ways. For example, it can be viewed as a language, serving as a tool for the development of knowledge and as an interpreter of social reality [2]. In recent times, the philosophy of mathematics has emerged as a specialized academic field, with its literature and community of scholars. The subject is now considered a branch of philosophy that employs a style of thinking and reasoning widely accepted by the philosophical community [3].

Mathematics, as a philosophy and a crucial component of education, serves as the foundation for the development of modern technology and plays an important role in various scientific disciplines. With the rapid development of globalization in the 21st century, a strong mastery of mathematics is essential. Therefore, it is imperative that the subject is taught at all levels of schooling, starting from elementary school [4].

In line with this goal, the Ministry of Education and Culture has developed innovative curriculums for each track, level, and type of educational institution. The most recent curriculum, known as the 2013 curriculum, aims to improve the quality of education by producing creative graduates who possess the skills to face the challenges of the future [5]. In today's world, creativity is a key attribute that is essential for development. The result of a survey conducted by Salmon revealed that 25 % of organizations with more than 100 staff provide training on creativity [6]. This means creative thinking skills are needed for success in today's workforce. Therefore, it is imperative that future generations be equipped with the ability to think creatively, allowing them to adapt to the ever-changing times.

Developing the ability to think creatively is a crucial aspect of education, particularly in the field of mathematics. Creative thinking skills have been shown to have a positive influence on students' understanding of mathematical concepts [7]. Therefore, mathematics education needs to be viewed as an opportunity for fostering creativity [8].

This notion is supported by numerous experts who have studied the subject of students' creative thinking abilities. For example, De Bono identified four levels of achievement in the development of creative thinking skills, namely thinking awareness, thinking observation, thinking strategies, and reflection on thinking [9].

Creative thinking is a product of both divergent and convergent thinking processes [10]. As reported by Runco and Acar [11], divergent thinking allows for the generation of novel ideas. Study results indicated that divergent thinking interacts with convergent thinking in the context of scientific creativity [12], which is a critical aspect of mathematics. Therefore, creative mathematical problem-solving requires the use of other divergent and convergent thinking strategies.

Considering the crucial nature of creative thinking skills, it is not surprising that numerous experts have conducted studies on this topic. Several renowned experts have contributed significantly to the field of international mathematics education and have served as a reference for others. Examples of such experts include Siswono [9], Runco and Acar [11], Paul [13], Leung and Silver [14], and Leung [15].

Despite the significance of creative thinking skills, there are still several contradictory cases suggesting that students are likely to lack proficiency in this area. It has been observed that the creative thinking abilities of elementary and middle school students in mathematics remain in a low category, with a percentage of 46.67 %. In this study, this ability was measured using an open-ended test designed to provide a clearer description of the creative thinking process. When asked questions that emphasized the number of correct answers and strategies for solving problems, most students responded with only one strategy. This finding suggests that students have limited abilities in terms of flexible mathematical creative thinking [16].

Similarly, the study conducted by Andiyana, Maya, and Hidayat, measured the ability to think creatively in the context of solid geometry. It was discovered that most students were unable to find the surface area of a pyramid because they either forgot or did not know the formula. This emphasizes the importance of being able to find answers in a different way or using new ideas in mathematical creative thinking. Unfortunately, the lack of originality in the students’ thinking led to poor performance on the questions [17]. In order to develop students' mathematical creative thinking skills, it is crucial to help them develop their existing abilities. One of the ways to achieve this goal is by instilling in students the value of self-regulated learning. This is because it has been established that there is a significant relationship between mathematical creative thinking skills and self-regulated learning [18]. Specifically, self-regulated learning has a positive effect of 85.4 % on mathematical creative thinking skills [19].

Self-regulated learning is an important individual factor that affects students' learning. It can be defined as the ability to demonstrate a sense of responsibility in regulating, self-disciplining, and developing one's learning abilities independently. Ultimately, students' self-regulated learning can be used as a benchmark for achieving good academic performance [20].

As reported by Liebendörfer, Kempen, and Schukajlow [21], self-regulated learning has garnered considerable attention since 1980. It refers to a learning process influenced by a person's thoughts, feelings, strategies, and behaviors that are oriented toward achieving goals [22]. In other words, self-regulated learning enables students to combine academic learning with self-control, thereby enhancing their motivation to learn in order to achieve learning goals independently, take self-responsibility in learning, and build their unique learning goals. Consequently, students do not feel constrained by lessons from teachers and feel more flexible and better equipped to learn independently.

This is evidenced by the discovery of Kurnia and Warmi, stating that the average score of students' self-regulated learning was only 27.6 % out of the maximum percentage of 100 %. The students’ learning plans were relatively low, at 26.55 %, leading to poor achievements, which were only at 28.53 %. The evaluation of their learning outcomes was also low at 26.8 %, which led to low achievements [23].

Based on the importance of mathematical creative thinking skills and their association with self-regulated learning, a study related to this scenario was deemed necessary. Relevant studies explored the relationship between mathematical creative thinking ability and various factors such as self-efficacy [24], self-confidence [25], self-concept [26], multiple representations and mathematical creativity [27], reasoning ability and creative thinking [28], problem-solving and creativity [29], critical, reflective, and creative thinking [30], as well as self-regulated learning and academic motivation [31]. Despite these investigations, there is currently no published paper that specifically investigates the ability to think creatively in mathematics within the context of self-regulated learning. The purpose of this study is to describe the mathematical creative thinking ability of students within the framework of self-regulated learning. The results are expected to offer a valuable and novel resource for investigations in the field of mathematics education.

## 2 Methods

A qualitative approach was employed using a case study design, then an in-depth analysis of a particular event, activity, or process was conducted based on one or more individuals [32]. The analysis focused on students’ mathematical creative thinking ability taking into account their self-regulated learning.

Data were collected through various means including a test, questionnaires, and interviews. The test was conducted to obtain data on students' mathematical creative thinking abilities. Questionnaires were used to gather information on the students’ self-regulated learning, while written data in the form of statements related to the answers provided to the test items were collected using an introductory interview questionnaire. Furthermore, an interview guideline was used to obtain more in-depth data on the reasons behind the students' answers to the questions given. This study has been reviewed by the Ethics Committee of the Universitas Islam Negeri Sultan Syarif Kasim Riau. Based on their decision, the investigation adheres to ethical standards, but before the study was conducted, informed consent was obtained from all participants.

To ensure the reliability of the findings, all instruments used, including test items, questionnaire sheets, and interview guidelines, were developed. The questions for measuring mathematical creative thinking ability were prepared based on indicators such as 1) thinking about more than one answer (fluency), 2) looking for several alternatives or different directions (flexibility), 3) thinking about unusual ways (authenticity), and 4) adding or breaking down the details of an idea, object, or situation to make it more interesting (elaboration) [19,33,34]. Each question represented one indicator, and for mathematical creative thinking ability test questions to be valid, reliable, discriminating, and appropriately challenging, it needs to meet certain criteria. In terms of validity, a test item is considered valid if the value of $r_{count}\geq r_{table}$, and this can be calculated using the following formula [35]:

$$r_{count}=\frac{NƩXY-\left(ƩX\right)\left(ƩY\right)}{\sqrt{\left\{NƩX^{2}-{\left(ƩX\right)}^{2}\right\}\;\left\{NƩY^{2}-{\left(ƩY\right)}^{2}\right\}}}$$

Where:

$r_{count}$ = count correlation coefficient between the score per item (X) and the total score (Y).

ƩX = total score of X.

ƩY = total score of Y.

N = the number of students

ƩXY = the number of times X is multiplied by Y.

$X^{2}$ = the square of X.

$Y^{2}$ = the square of Y.

The reliability, level of discrimination indices, and difficulty level of the test items used were determined based on the guidelines presented by Lestari and Yudhanegara [36], as seen in Table 1 below.

**Table 1:** Reliability level, difficulty level, and level of discrimination indices.

Reliability Level		Difficulty Level		Level of Discrimination Indices	
Value	Interpretation	Value	Interpretation	Value	Interpretation
0.90 < r < 1.00	Very good	0.70 < DL ≤ 1.00	Very good	LDI = 0.00	Too hard
0.70 < r < 0.90	Good	0.40 < DL ≤ 0.70	Good	0.00 < LDI ≤0.30	hard
0.40 < r < 0.70	Moderate	0.20 < DL ≤ 0.40	Moderate	0.30 < LDI ≤0.70	Moderate
0.20 < r < 0.40	Bad	0.00 ≤ DL ≤ 0.20	Bad	0.70 < LDI <1.00	Easy
r ≤ 0.20	Very bad	DL < 0.00	Very bad	LDI = 1.00	Too easy

Notes.

r = reliability values.

LDI = level of discrimination indices.

DL = difficulty level.

The $r_{table}$ the calculation result for the validity of the test questions is 0.374. Data was obtained based on the data processing results, as shown in Table 2 below.

**Table 2:** The summary of development results of the questions.

Item No	Validity Level	Reliability Level	Difficulty Level	Level of Discrimination Indices
1	Valid (0.50)	Moderate (0.76)	Moderate (0.64)	Moderate (0.25)
2	Valid (0.71)		Moderate (0.62)	Moderate (0.34)
3	Valid (0.77)		Moderate (0.56)	Good (0.45)
4	Valid (0.77)		Moderate (0.55)	Moderate (0.34)

**Fig. 1:**
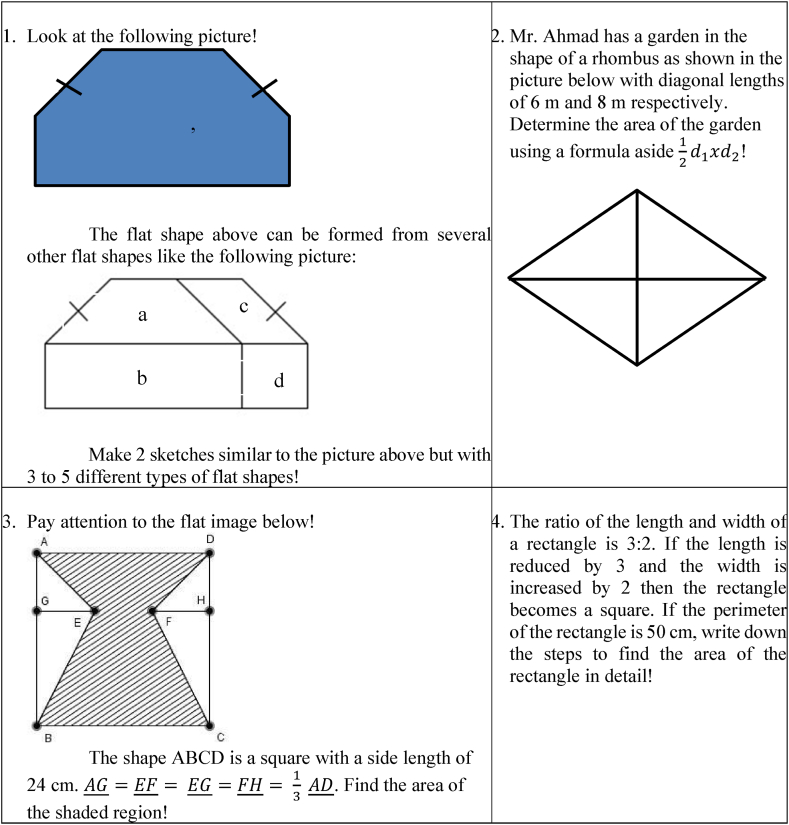
Creative thinking ability test questions.

The questions considered can be seen in the following Fig. 1.

Question number 1 measures the ability to think about more than one answer (fluency), while question number 2 assesses the ability to look for several alternatives or different directions (flexibility). Question number 3 is related to thinking about unusual ways (authenticity), and question number 4 measures the ability to add or break down the details of an idea, object, or situation to make it more interesting (elaboration).

The questionnaires for self-regulated learning were prepared using 13 indicators, which were divided into two components. These indicators represent a combination of concepts proposed by Lestari and Yudhanegara [36], as well as Hendriana, Rohaeti, and Sumarmo [37]. The details of these indicators can be seen in Table 3 below.

**Table 3:** Indicators of self-regulated learning.

Components	Indicators	Total of Statements in The Questionnaire
The process of obtaining information	1. diagnosing learning needs	2
	2. having the initiative to learn	2
	3. being able to solve problems	1
	4. seeing difficulties as challenges	1
	5. evaluating learning processes and outcomes	1
	6. utilizing and searching for relevant sources	3
	7. having the ability to self-determination	2
	8. monitoring, organizing, and controlling learning	2
	9. making your own decisions	1
Transformation process	1. able to hold back	1
	2. setting the learning goals or targets	2
	3. choosing and implementing learning strategies	3
	*4. self-efficacy*	**2**
**Total of Statements in The Questionnaire**		**23**

The questionnaire consisted of 23 statements that students were required to select based on their responses to the questions related to creative thinking abilities. The validity and reliability of these statements were determined using the same formula and calculation method as the ones used for the test questions. The results indicated that the 23 statements were in the valid category and demonstrated good reliability (0.87).

The investigation was performed during the second semester at a State Junior High School in Riau, involving a total of 28 VIII-C students. Research subjects were selected by cluster random sampling (classes were considered clusters) with a lottery technique. This selection of class VIII students was carried out because the prepared learning material was taught for class VIII level and the students had received the material. The questions of the test instrument were contained in the learning material and were tested on the class VIII students. Before selecting research subjects, the researchers ensured that the five classes consisted of students with diverse academic abilities. This was for the aim that the research results regarding students’ answers in solving creative thinking ability test questions would be more varied and reflect the many possible answers that could be given by the subjects. Therefore, researchers have conducted interviews with mathematics teachers and deputy principals to ensure that no single class is a superior class, and each class consists of students with varying academic abilities.

These participants completed a self-regulated learning questionnaire and a mathematical creative thinking ability test. Consequently, the data obtained from the questionnaires and answer sheets were examined and processed.

The answers provided in response to the creative thinking ability test questions were evaluated using a scoring system ranging from 0 to 4. The scoring criteria were developed and modified based on the four specific indicators of creative thinking ability being studied [38]. Each indicator had distinct criteria, leading to varied expectations for different score values. The more detailed scoring guidelines could be seen in Table 4.

**Table 4:** Guidelines for scoring mathematical creative thinking ability.

Indicators	Score	Students' Responses to Questions or Problems
Fluency	0	Do not answer or provide ideas that are not relevant to the problem.
	1	Give an idea that is not relevant to solving the problem.
	2	Give a relevant idea but the answer is still wrong.
	3	Give more than one relevant idea but the answer is still wrong.
	4	Provide more than one relevant idea, as well as a correct and clear solution.
Flexibility	0	Not answering or providing an answer in one way or more but all wrong.
	1	Answering in only one way, which is wrong.
	2	Give answers in one way, the calculation process and the results are correct.
	3	Give answers in more than one way (various) but the results are wrong because there is an error in the calculation process.
	4	Give answers in more than one way (various), and the calculation process of the results is correct.
Authenticity	0	Do not provide an answer or give the wrong answer.
	1	Give an answer in their way but cannot be understood.
	2	Give answers in their way, the calculation process is directed but not finished.
	3	Give answers in their way but the results are wrong because there is an error in the calculation process.
	4	Give answers in their way, the calculation process, and the results are correct.
Elaboration	0	Do not answer or give the wrong answer.
	1	There is an error in the answer and it is not accompanied by details.
	2	There is an error in the answer but it is accompanied by less detail.
	3	There is an error in the answer but it is accompanied by detailed details.
	4	Give correct and detailed answers.

Students’ answers in relation to the self-regulated learning questionnaires were examined based on the type of statement, distinguishing between negative and positive statements. Negative statements were assigned a score of 1 for the answer choice A (Always), 2 for answer choice O (Often), 3 for choice S (Sometimes), and 4 for choice N (Never). Meanwhile, positive statements followed the reverse scoring pattern, and the questionnaire used for this purpose consisted of 11 positive and 12 negative statements, which were distributed among 13 indicators.

The total average and standard deviation of the scores were obtained on the self-regulated learning questionnaire and were computed. Subsequently, each student was categorized as high, medium, and low self-regulated learning based on their respective scores with the concept of standard deviation [39], as shown in Table 5.

**Table 5:** Criteria for grouping students’ self-regulated learning.$\underline{x}=Average\;total\;score\;of\;self-regulated\;learning=64.07$

Self Regulated Learning Criteria	The score of Self Regulated Learning Criteria	Group	No of Students
$x\geq \left(\underline{x}+SD\right)$	Score ≥73.93	High	5
$\left(\underline{x}-SD\right)<x<\left(\underline{x}+SD\right)$	54.22≤ Score <73.93	Moderate	20
$x\leq \left(\underline{x}-SD\right)$	Score <54.22	Low	3

SD= Standard deviation of the total score of self-regulated learning = 9.86

To facilitate participant identification, each individual was assigned a code based on the alphabetical order of their names. For example, students with first name order were designated as S-1, and this pattern continued sequentially until the 28th name, which was coded as S-28.

Out of the 28 participants, the data of 9 were analyzed in greater depth. These 9 participants were selected based on their varied responses to the given mathematical creative thinking ability test questions. Specifically, 3 participants were selected from the group of students with high self-regulated learning, namely S-11, S-19, and S-26 codes. Another 3 individuals were selected from the group of students with moderate self-regulated learning (codes S-2, S-5, and S-7). The final 3 participants were taken from students with low self-regulated learning (codes S-18, S-24, and S-27).

Subsequently, the examiner administered an introductory questionnaire to the nine selected participants before the interview. This questionnaire was carefully designed to anticipate potential responses to questions and included statements related to common errors made by students while providing the answers. The questionnaire employed a Guttman scale with two options, namely (1) “yes” and (2) “no”, to provide a framework for conducting the subsequent interviews.

After analyzing the correlation between the creative thinking ability score and the student's responses to the introductory questionnaire, a direct interview was conducted with the 9 selected participants using a semi-structured interview guide as a reference. The guide has been defined, compiled, and prepared beforehand [40], and it contained questions intended to explore the students' mathematical creative thinking abilities obtained through the test. Notably, semi-structured interview guides are commonly used in the qualitative study [41,42]. This means that the questions were not asked spontaneously during the interview. The interviews were conducted with 3 students representing each self-regulated learning category in order to obtain more in-depth information about their abilities.

This study was conducted with several considerations to ensure the comfort and ease of the participants. The examiner carefully selected the timing of the data collection in the morning and provided a comfortable and convenient location within the school premises. Communication with the students was carried out using clear and understandable language while avoiding harsh or intimidating words during the process. Additionally, the school's support was ensured in order to facilitate the smooth conduct of the activities.

The collected data were analyzed in three distinct stages, namely data reduction, data presentation, and the conclusion [43].

## 3 Results and discussion

### 3.1 Results

#### 3.1.1 Description of quantitative data on students' mathematical creative thinking ability

##### 3.1.1.1 The overall mathematical thinking ability of the students

Table 6 provides a comprehensive summary of the student's scores for each item based on their responses to mathematical creative thinking skills questions.

**Table 6:** The average score of students’ mathematical creative thinking ability on each item.

No.	Items Number	Maximum Score	Average Score	Percentage
1	1	4	2.71	67.86
2	2	4	1.89	47.32
3	3	4	2.61	65.18
4	4	4	0.32	8.04
Total		16	7.54	47.06
Overall Average			1.88	45.09

According to the data presented in Tables 6 and it was observed that students had a low level of mastery of mathematical creative thinking ability. This is evident from the overall average score of 1.88, which is significantly lower than the maximum ideal score of 4. The data suggests that students were able to master only 45.09 % of this particular ability.

On a more detailed examination, it was observed that students struggled to master the answers to question number 4, as only 20 out of 28 students scored 0 (Table 7). This implies that students had difficulty analyzing and deconstructing the details of an idea, object, or situation. On the other hand, they fared relatively well in answering item number 1, with 21 out of 28 students receiving 3 points and 2 respondents scoring 4 points. This infers that the students were able to think critically and generate multiple answers.

**Table 7:** The overall results of mathematical creative thinking skills tests.

Number of Students Obtaining the Score	Item Number			
	1	2	3	4
4	2	2	9	0
3	21	13	12	0
2	1	2	0	1
1	3	2	1	7
0	1	9	6	20

##### 3.1.1.2 Students' mathematical creative thinking ability in terms of self-regulated learning

The data on students' mathematical creative thinking abilities based on self-regulated learning were classified into three categories, namely respondents with high, medium, and low self-regulated learning. The details of the score can be seen in the following three tables.

Table 8 clearly indicates that students with high self-regulated learning consistently scored higher on mathematical creative thinking skills than their peers with moderate and low self-regulated learning. The student's highest level of mastery was observed for item number 3, which required thinking in unusual ways. Conversely, the lowest mastery level was recorded for item number 4, which involved analyzing and breaking down the details of an idea, object, or situation to make it more interesting. This trend was observed among students with moderate self-regulated learning. However, the results were different for students with low self-regulated learning. They appeared to be more proficient in item number 1, which required thinking of multiple answers. More detailed data can be found in Table 9 below.

**Table 8:** The average score of students’ mathematical creative thinking skills in terms of self-regulated learning.$\underline{x}=Average\;score\;of\;self-regulated\;learning$%=Percentageoftheaveragescoreofself−regulatedlearning

No	Indicators of Creative Thinking	Maximum Score	Level of Self-Regulated Learning					
			High		Moderate		Low	
			$\underline{x}$	%	$\underline{x}$	%	$\underline{x}$	%
1	Thinking of more than one answer	4	3.2	80	2.67	66	2.4	60
2	Looking for alternatives or different directions	4	2	50	1.89	47	1.8	45
3	Thinking of an unusual way	4	3.4	85	2.72	68	1.4	35
4	Planning the details of an idea, object, or situation to be more interesting	4	0.8	20	0.22	6	0.2	5
Average			9.4	58	7.5	46	5.8	36

**Table 9:** The distribution of scores of students' mathematical creative thinking ability in terms of self-regulated learning.

Level of Self-Regulated Learning	Number of Students Obtaining the Score	Item Number			
		1	2	3	4
High	4	1	0	2	0
	3	4	3	3	0
	2	0	0	0	1
	1	0	1	0	2
	0	0	1	0	2
Total		5			
Moderate	4	1	2	7	0
	3	14	8	7	0
	2	0	1	0	0
	1	2	0	0	4
	0	1	7	4	14
Total		18			
Low	4	0	0	0	0
	3	3	2	2	0
	2	1	1	0	0
	1	1	1	1	1
	0	0	1	2	4
Total		5			

#### 3.1.2 Description of qualitative data on students' mathematical creative thinking ability

##### 3.1.2.1 The overall mathematical creative thinking ability of the students

In addition to collecting data on students' mathematical creative thinking abilities through test scores, direct interviews were also conducted with selected students to gain further insight into the difficulties and errors experienced while answering the questions. Prior to the interview, a questionnaire was administered in order to gain preliminary knowledge about the student's responses and to serve as a guide in case the interview results differed slightly from the predicted outcome. A summary of the data obtained through these interviews can be found in Table 10 below.

**Table 10:** Details of the overall mathematical creative thinking ability of students.

Item Number	Details of Students' Mathematical Creative Thinking Ability	Types of Mistakes
1	Most students were less able to solve questions and to meet the criteria to think of more than one answer.	A small number of students made many mistakes in drawing a flat shape by not looking at the characteristics or features of each shape and also could not make 3 or more different shapes that were asked about.
2	Most students were less able to solve questions and meet the criteria for finding alternative answers or different directions.	Students made many mistakes in the way they chose to solve the questions that were not in accordance with the questions asked.
3	Most students were less able to solve questions and meet the criteria of thinking in unusual ways.	Students made many mistakes in the operation of algebra.
4	Most students were unable to solve problems and to meet the criteria of breaking down the details of an idea, object, or situation to be more interesting.	A lot of students could not describe what information was in the questions and could not specify the details of the answers they made.

##### 3.1.2.2 Students' mathematical creative thinking ability in terms of self-regulated learning

In addition to analyzing the data comprehensively, information on every self-regulated learning cluster was acquired. This data is presented in the following Table 11, Table 12, Table 13.

**Table 11:** Details of students' mathematical creative thinking ability with high self-regulated learning.

Study Subject	Findings	Remarks
Students with 11th name order (Code: S-11)	Students were not maximal in breaking down an idea, object, or situation.	Students were unable to describe the steps of the answer in complete detail, implying that they cannot solve the problem in item number 4 perfectly.
Students with 19th name order (Code: S-19)	Students were lacking in the ability to find many alternative answers and did not have good skills in breaking down an idea, object, or situation.	Students were unable to formulate the information contained in the item and describe the steps of the answer in detail. Therefore, they can not solve the problem in item number 4.
Students with 26th name order (Code: S-26)	Students were less capable in the ability to find many alternative answers and did not have good skills in breaking down an idea, object, or situation.	Students were not careful in understanding the questions given since that was the method used in solving item number 2. Also, they were unable to solve the questions given in item number 4.

**Table 12:** Details of students' mathematical creative thinking ability with moderate self-regulated learning.

Study Subject	Findings	Remarks
Students with 2nd name order (Code: S-2)	Students were unable to describe in detail an idea, object, or situation.	Students were unable to answer the given questions completely.
Students with 5th name order (Code: S-5)	Students were lacking in the ability to think of more than one answer and to specify in detail an idea, object, or situation.	In solving item number 1, the student could not make 3 to 5 different flat shapes in the sketch.
Students with 7th name order (Code: S-7)	Students were less able to describe in detail an idea, object, or situation.	Students were unable to formulate the information in the question and describe the steps of the answer in complete detail. Therefore, they can not solve the problem in item number 4.

**Table 13:** Details of students' mathematical creative thinking ability with low self-regulated learning.

Study Subject	Results	Remarks
Students with 18th name order (Code: S-18)	Students were less able to think about unusual ways and detail an idea, object, or situation.	Students were not careful in the operation of item number 3 and could not solve the problem well in item number 4.
Students with 24th name order (Code: S-24)	Students were not able to find alternative answers and specify in detail an idea, object, or situation.	Students were not able to find the correct method in item number 2 and were unable to solve the problem properly because they did not answer the question given in number 4.
Students with 27th name order (Code: S-27)	Students were not able to think of unusual ways and specify in detail an idea, object, or situation.	Students were unable to answer the items given to them.

The following is an example of a conversation during an interview with subject S-19:

S-19: "Determine the area of the garden using a formula other than the formula $\frac{1}{2}d_{1}{x\;d}_{2}$!"

Researcher: "Can you tell how you got the answer to question number 2?"

S-19: "Diagonal minus diagonal divided by two, 6 minus 8 divided by 2, -2 divided by 2 results −1."

Researcher: "Where did you get references like this?"

S-19: "Books and teachers."

Researcher: "Are you sure your answer is correct?"

S-19: "Sure."

In the introductory questionnaire for the interview, subject S-19 responded negatively to the statement regarding the difficulty in obtaining an answer for question number 2". Through an examination of the data collected from the questionnaire responses and the subsequent conversation between the researcher and subject S-19, it became evident that respondent understood question number 2 (related to the indicator of looking for many alternatives or different directions). S-19 expressed a desire to identify different alternatives but encountered challenges in successfully identifying and documenting answers in accordance with the given questions. This led to the conclusion that S-19 showed limited proficiency in generating different alternative answers.

The following is an example of a conversation during an interview with subject S-5:

Researcher: "How did you get the idea of this picture?"

S-5: (silence).

Researcher: "From the answers you wrote, how many flat shapes did you draw?"

S-5: "There are 2."

Researcher: "What?"

S-5: "Triangle and rectangle."

Researcher: "This one?" (While pointing to the 2nd sketch which is point d).

S-5: “Trapezium.”

Researcher: "From what you made, are there any other answers that can be made apart from your answer?"

S-5: "Yes."

Researcher: "That means you can draw another sketch besides the one you did?"

S-5: “Yes sir.”

The interview conducted with S-5 showed that the subject was able to identify the names of the shapes presented and could generate alternative answers. It was observed that the respondent only produced sketches involving 2 flat shapes, despite the instruction specifying the creation of sketches with 3–5 planes of different types. Based on this scenario, it was concluded that S-5 lacked a comprehensive understanding of the given questions. This observation was further supported by the difficulty faced by the respondent in sketching the two-dimensional shape for question number one, as indicated in the introductory questionnaire (associated with the indicator of generating multiple answers conveniently).

The following is an example of a conversation during an interview with subject S-24:

Researcher: "Can you mention what information is in problem number 4?"

S-24: “I do not know sir.”

Researcher: "So you cannot solve this problem?"

S-24: “Yes sir.”

During the interview conducted with subject S-24, it became apparent that the student encountered challenges in completing question number 4, which involved the indicator of adding or elaborating details to enhance the interest in an idea, object, or situation. This observation was further substantiated by the questionnaire statement indicating that there was difficulty in obtaining information about the problem and describing the steps involved. Consequently, it could be concluded that S-24 exhibited limited proficiency in elaborating on ideas, objects, or situations.

## 4 Discussion

### 4.1 The overall mathematical creative thinking ability of students

The results of this study indicated that students presented diverse solutions when solving problems designed to assess their mathematical creative thinking skills. This outcome aligns with the nature of mathematical creative thinking, which requires students to generate different methods or steps and even varied answers. It was observed that the range of methods and mathematical responses provided by students correlated significantly with their understanding of the rectangular solid (geometry).

Based on the results of the data analysis, it can be concluded that the respondents varied in their ability to solve solid (geometry) questions. The majority of students were able to think of more than one answer but struggled with breaking down the details of an idea, object, or situation. This is due to a tendency of jumping to conclusions without fully considering all relevant factors.

To address this issue, the researcher could consider implementing strategies that encourage students to slow down and engage in more detailed analysis. For example, students could be given explicit instructions to take more time when considering each question and to carefully evaluate all possible options before making a final decision. Additionally, the researcher could provide students with more practice opportunities that require them to analyze complex problems in detail, rather than simply generating a list of potential answers. With practice and guidance, students may develop stronger analytical skills and become more adept at breaking down complex problems.

The results showed that students from a State Junior High School in Riau had a relatively low average ability level, achieving a score of 45.09 %. This ability was measured through various components, including fluency, flexibility, authenticity, and elaboration. The fluency component, which refers to generating multiple answers, scored the highest at 67.86 %, followed by authenticity at 65.18 %, and flexibility at 47.32 %. However, the elaboration component, which involves breaking down the details of an idea, object, or situation to make it more interesting, scored the lowest at 8.04 %. This is consistent with a previous study titled “An Analysis of Senior High School Student's Mathematical Creative Thinking Ability Regarded Geometry”. In the study, the maximum score for mathematical creative thinking ability was 100, with fluency, flexibility, authenticity, and elaboration accounting for 30, 25, 25, and 20 points, respectively. From these scores, the elaboration component was found to be the least rated indicator compared to others [33].

A study conducted by Andiyana, Maya, and Hidayat, entitled "Analysis of Mathematical Creative Thinking Ability of Junior High School Students in Solid (Geometry)" similarly found that students in Ngamprah Village had low mathematical creative thinking ability in solid material, scoring an average of 51 %. However, the distribution of percentages for each component varied, with flexibility scoring the highest at 87.5 %, followed by fluency at 56.3 %, elaboration at 50 %, and originality at the lowest at 12.5 %. This difference can be attributed to the fact that the flexibility component had the highest score, while originality had the lowest [17].

### 4.2 The students' mathematical creative thinking ability in terms of self-regulated learning

The results of this study indicated that the students exhibited varying levels of mathematical creative thinking skills based on the answers provided. The variation in the completion level of the quadrangles was caused by several factors, one of which was students' self-regulated learning. As reported by Long, learning is a cognitive process influenced by several factors, such as individual circumstances, prior knowledge, attitudes, individual views, content, and presentation methods. One important sub-factor of individual circumstances that influence learning is self-regulated learning [44]. This was supported by Bungsu, Vilardi, Akbar, and Bernard, which found a significant positive effect of self-regulated learning on mathematics learning outcomes. Self-regulated learning contributed 16 % to mathematical learning outcomes, while the remaining 84 % was influenced by other variables. This implied that understanding the students’ self-regulated learning is very important [45].

The analysis of a self-regulated learning questionnaire using 13 indicators helps in classifying and drawing conclusions. It was found that students with high self-regulated learning paid more attention to learning initiatives. This was evident in the data obtained, showing that the indicator of having initiative in learning received a higher value than the other indicators. Additionally, it is important to note that evaluating the students’ learning processes and outcomes was another crucial indicator that needs to be considered. However, it received a lower average score compared to the other indicators.

Overall, the level of self-regulated learning greatly affected the students’ mathematical creative thinking ability. From the data analysis results, it was observed that students with high self-regulated learning tended to have high mathematical creative thinking skills. The discussion of each category of self-regulated learning can be stated as follows.

### 4.3 Mathematical creative thinking ability obtained by high self regulated learning subjects (S-11, S-19, and S-26)

Based on the findings, it was found that students S-11, S-19, and S-26 were able to understand the questions for the indicators to think of more than one answer, but struggled to draw optimal sketches. For example, there was no noticeable difference in the sketches made by the students for squares and rectangles. During the interview, the students were able to mention all the shapes of planes they had constructed but tended to forget about the properties and characteristics of the planes when attempting to draw them on paper. Therefore, it can be concluded that the students had the ability to provide multiple answers, but faced difficulties when it came to sketching.

Although the aforementioned three students were able to provide different alternatives in response to the indicator of searching for multiple directions, they were not proficient in documenting the appropriate steps. In terms of the indicator of thinking in unusual ways, these students were able to construct planes based on the given sketches but were inaccurate in executing the steps, leading to subpar answers. Finally, the data revealed that while the students were capable of understanding the questions and providing information during the interview as an indicator of breaking down the details of an idea, they struggled to break down the steps in solving the given problems on the worksheet. Despite being in the highly self-regulated learning category, these students were still deficient in the indicator of breaking down the details of an idea compared to the other three indicators of mathematical creative thinking, namely thinking of multiple answers, searching for different alternatives, and thinking in unusual ways.

The low ability of students with high self-regulated learning in detailing an idea, an object or a situation is in line with the characteristic concept of self-regulated learning itself. One of the self-regulated learning characteristics related to cognitive factors is being able to organize [46,47]. Meanwhile, detailing is the opposite of organizing (making one). So, it is natural that the higher a student does self-regulated learning, the more difficult the student to elaborate it. This is because students’ organizational abilities will be better. As stated by Stebner et al., by fostering self-regulated learning, the students will be increasingly able to organize their learning consciously [48].

### 4.4 Mathematical creative thinking ability obtained by moderate self-regulated learning subjects (S-2, S-5, and S-7)

The findings of this study revealed that although students in the category S-2, S-5, and S-7 were able to understand the questions for the indicators to think of more than one answer, they were still inaccurate in describing the sketch. This was exemplified by the similarities of sketches they made for squares and rectangles. Meanwhile, these students were able to mention all the shapes that had been made during the interview, they tended to forget about the properties and characteristics of planes when drawing on paper. This implied that these respondents were able to provide more than one answer but were inaccurate in sketching. This issue was also observed in students with high self-regulated learning.

Moreover, the data analysis revealed that these three categories of students provided answers with varied alternatives for the indicator of looking for different directions, but were not maximal in writing the appropriate steps. For the indicator of thinking in unusual ways, these students were able to construct the planes on the given sketches, but were inaccurate in operating the steps, implying that their answers were not as good as expected. Regarding the indicator of breaking down the details of an idea, the respondents understood the questions and were confident in what they were doing during the interview. However, they were unable to break down the steps in solving the problems given on the worksheet. Based on this discussion, it could be concluded that the moderate self-regulated learning students lacked the indicator of breaking down the details of an idea compared to other indicators. While they were able to think of more than one answer, look for different alternative answers, and think in unusual ways, they still needed to improve their skills in breaking down the details of an idea, object, or situation.

### 4.5 Mathematical creative thinking ability obtained by low self-regulated learning subjects (S-18, S-24, and S-27)

After analyzing the data, it was discovered that students S-18, S-24, and S-27, who were classified as having low self-regulated learning, were able to generate multiple solutions for the first stage of mathematical problems. However, they struggled with drawing the appropriate sketches. During the interview, the respondents accurately identified the shapes but were unable to provide any additional information beyond the answers already produced. This means that as these three categories of students were able to provide more than one answer, they faced difficulties in creating and matching the intended sketches.

Regarding the indicator of looking for many alternatives or different directions, this study found that the three students were able to provide different alternative answers but the steps made did not lead to the desired answer. Furthermore, for the indicator of thinking in an unusual way, these respondents were unable to provide answers to the given problems. During the interview, the students admitted they had not encountered such questions before. This indicated that it was their first time encountering such problems. Concerning the indicator of breaking down the details of an idea, these three students were able to understand the questions and provide information on the problem. However, they were not able to accurately write down the details of the answer in the worksheet. Based on this discussion, it can be concluded that students with low self-regulated learning were less able to think of more than one answer and look for different alternative solutions. They were also lacking in the indicators of thinking in unusual ways and breaking down the details of an idea, object, or situation.

From the data analysis results, it was found that most students had low proficiency in breaking down the details of an idea, object, or situation in order to make it more captivating. This deficiency was due to many factors, one of them being the level of self-regulated learning exhibited by the students. The results demonstrated that respondents with high self-regulated learning had better creative thinking abilities than those with moderate and low self-regulated learning.

These results were in line with a study conducted by Meiliana and Aripin in 2019 [49], which explored the relationship between creative thinking and self-regulated learning among junior high school students. Meiliana and Usman argued that students who had high creative thinking skills were also likely to possess a strong sense of self-regulated learning. However, in this current study, the lowest score for mathematical creative thinking ability was observed in the authenticity component, specifically in the indicator of thinking in an unusual way [49]. These findings diverged from the Meiliana and Usman study, in which the students tended to exhibit low creative thinking skills in the elaboration component. Meanwhile, the authenticity component with the indicator of thinking in an unusual way was more likely to be high. This discrepancy occurred because of the differences in students' creative thinking abilities. Notably, the data collection activity was conducted in a comfortable and stress-free environment that supported good communication and interaction between the examiners and students.

However, certain conditions are accounting for the differences in the characteristics of mathematical creative thinking ability. One possible explanation for the discrepancy between the findings of this study and that of Meiliana and Aripin [49] is the difference in locations. Specifically, this current study was conducted at a State Junior High School in Riau, Indonesia, while Meiliana and Aripin carried out their investigation at 1 Junior High School in Margaasih, Cimahi. Additionally, the differences in materials used can also contribute to the variation in results. In this study, rectangular materials were utilized while Meiliana and Aripin employed Relations and Functions.

Furthermore, this research found that students’ biggest weakness in creative thinking lies in the indicator of breaking down the details of an idea, object, or situation in order to make it more captivating. Meanwhile, the opposing word of detailing is organizing. And, organizing is one of the characteristics of self-regulated learning. Therefore, these findings open a gap for future researchers to study this matter in more depth.

This study has certain limitations, one of which pertains to the limited number of participants. Out of 28 participants, only 9 were selected for in-depth qualitative analysis. This limitation is also evident in previous studies, where only nine participants were interviewed [42], or four out of 60 students were selected for qualitative analysis [50]. Similarly, other qualitative studies have also considered a small number of respondents, such as 3 [21], or 20 participants [51].

## 5 Conclusion

After analyzing the data and discussing the mathematical creative thinking skills of students in terms of self-regulated learning, the following conclusions were drawn, namely (1) the students at a State Junior High School in Riau showed low levels of mathematical creative thinking skills, with the lowest ability being the breakdown of details to make an idea, object, or situation more interesting, while the highest was thinking about multiple answers. (2) The students' mathematical creative thinking skills in terms of self-regulated learning include (a) Students with high self-regulated learning had high creative thinking skills, they were able to think of multiple answers, consider different alternatives, and think in unusual ways. However, they had limitations in breaking down details to make an idea, object, or situation more interesting. (b) Students with moderate self-regulated learning had moderate creative thinking skills, as they were able to think of multiple answers and look for different alternatives. Moreover, they are deficient in thinking in unusual ways and breaking down details to make an idea, object, or situation more interesting. (c) Students with low self-regulated learning had low creative thinking skills, as they were sufficiently able to think of more than one answer and look for different alternatives. Unfortunately, they struggled with explaining and applying the desired method to the problem. Additionally, the students were less able to think in unusual ways and break down details to make an idea, object, or situation more interesting.

Based on the discussion of the results, several important considerations were made as follows.

1. The results of a study can be made more resourceful by involving more participants. Therefore, further studies need to include a larger number of participants in order to obtain more meaningful conclusions.
2. The strong correlation between high levels of self-regulated learning and mathematical creative thinking abilities presents an opportunity for studies aimed at improving self-regulated learning. Experimental studies implementing specific learning activities can be conducted to enhance the mathematical creative thinking skills of students.
3. Based on the abundance of theories and studies exploring the relationship between cognitive and affective abilities, it is important to note that studies focusing on the affective aspects of creative thinking abilities remain relatively scarce. Therefore, it is imperative for future studies to examine mathematical creative thinking abilities from the perspective of other affective aspects, leading to a more comprehensive understanding of the topic.

## Data availability

The data that support the result of this study are available upon request from the authors.

## Funding statement

The authors received no financial support or specific grant from any funding agency for the research, authorship, and publication of this article.

## CRediT authorship contribution statement

**Hayatun Nufus:** Writing – review & editing, Writing – original draft, Project administration, Methodology, Investigation, Funding acquisition, Formal analysis, Data curation, Conceptualization. **Ramon Muhandaz:** Writing – review & editing, Writing – original draft, Project administration, Methodology, Investigation, Funding acquisition, Formal analysis, Data curation, Conceptualization. **Hasanuddin:** Writing – review & editing, Writing – original draft, Validation, Resources, Methodology, Investigation, Formal analysis. **Erdawati Nurdin:** Writing – review & editing, Writing – original draft, Visualization, Validation, Resources, Methodology, Formal analysis, Data curation. **Rezi Ariawan:** Writing – review & editing, Writing – original draft, Visualization, Resources, Data curation. **Rira Jun Fineldi:** Writing – review & editing, Writing – original draft, Validation, Software, Resources, Project administration. **Isnaria Rizki Hayati:** Writing – review & editing, Writing – original draft, Visualization, Validation, Resources. **Dominikus David Biondi Situmorang:** Writing – review & editing, Writing – original draft, Visualization, Validation, Supervision, Resources, Methodology, Formal analysis.

## Declaration of competing interest

The authors declare the following financial interests/personal relationships which may be considered as potential competing interests: Dominikus David Biondi Situmorang has the position to declare himself as one of Associate Editors in this journal. However, this article has been handled by another unidentified Associate Editor and reviewed by the Reviewers in an objective and double-blind manner, according to applicable regulations from Elsevier, Heliyon, and Cell Press.
