# Supplementary material for: Analyzing the students’ mathematical creative thinking ability in terms of self-regulated learning: How do we find what we are looking for?
Source: Heliyon. 2024 Jan 20;10(3):e24871. doi: 10.1016/j.heliyon.2024.e24871 (PMC10838748; doi:10.1016/j.heliyon.2024.e24871)
Supplement: Multimedia component 1 [file mmc1.docx]

**QUESTIONS TEST OF MATHEMATICAL CREATIVE THINKING ABILITY**

Full Name :

School :

Class / Semester :

Date :

**Instruction:**

**1. Please write your complete identity on the answer sheet provided.**

**2. Work on the problems that you find easy first**

**3. Double-check your answers before submitting them to the teacher.**

1. Take a look at the following picture!

,

The flat shape above can be formed from several other flat shapes like the following picture:


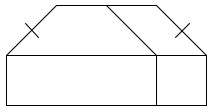


b

a

d

c

Make 2 sketches like the picture above but with 3 to 5 different types of flat shapes!

Answer:

............................................................................................................................................................................................................................................................................................................................................................................................................................................................................................................................................................................................................................................................................................................................................................................................................................................................................................................................................................................................................................................... .................................................................................................................................................................................................................................................................................................................................................................................................................................................................................................................................................................................................................................................................................................................................

............................................................................................................................................... .................................................................................................................................................................................................................................................................................................................................................................................................................................................................................................................................................................................................................................................................................................................................

1. Mr. Ahmad has a rhombus-shaped garden as shown in the picture with diagonal lengths of 6 m and 8 m respectively. Find the area of the garden using a formula other than the formula $\frac{1}{2}d_{1}xd_{2}$!

Answer:

.................................................................................................................................................................................................................................................................................................................................................................................................................................................................................................................................................................................................................................................................................................................................................................................................................................................................................................................................................................................................. ................................................................................................................................................................................................................................................................................................................................................................................................................................................................................................................................................................................................................................................................................................................................................................................................................................................................................................................................................................................................................................................

................................................................................................................................................................................................................................................................................................................................................................................................................................................................................................................................................................................................................................................................................................................................................................................................................................................................................................................................................................................................................................................

................................................................................................................................................................................................................................................................................................

1. Pay attention to the flat wake below!


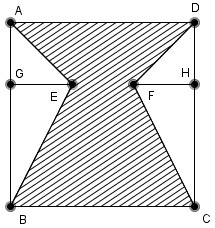


The shape ABCD is a square with a side length of 24 cm. $\bar{AG}=\bar{EF}= \bar{EG}=\bar{FH}= \frac{1}{3} \bar{AD}$. Find the area of ​​the shaded region!

Answer:

............................................................................................................................................................................................................................................................................................................................................................................................................................................................................................................................................................................................................................................................................................................................................................................................................................................................................................................................................................................................................................................... ................................................................................................................................................................................................................................................................................................................................................................................................................................................................................................................................................................................................................................................................................................................................................ ................................................................................................................................................

............................................................................................................................................... ................................................................................................................................................................................................................................................................................................................................................................................................................................................................................................................................................................................................................................................................................................................................................ ................................................................................................................................................

............................................................................................................................................... ................................................................................................................................................................................................................................................................................................................................................................................................................................................................................................................................................................................................

1. The ratio of length and width of a rectangle is 3: 2. If the length is reduced by 3 and the width is increased by 2, the rectangle becomes a square. If the perimeter of the rectangle is 50 cm, write down the steps to find the area of the square in detail and completely!

Answer:

............................................................................................................................................................................................................................................................................................................................................................................................................................................................................................................................................................................................................................................................................................................................................................................................................................................................................................................................................................................................................................................... ................................................................................................................................................................................................................................................................................................................................................................................................................................................................................................................................................................................................................................................................................................................................................................................................................................................................................................................................................................................................................................................................................................................................................................................................................................................................................................................................................................................................................................................................................................................................................................................................................................................

................................................................................................................................................................................................................................................................................................................................................................................................................................................................................................................................................................................................................................................................................................................................................................................................................................................................................................................................................................................................................................................................................................................................................................................................................................................................................................................................................................................................................................................................................................................................................................................................................................................

................................................................................................................................................................................................................................................................................................................................................................................................................................................

**HAVE A GREAT TIME DOING IT ☺**
