# Supplementary material for: Analyzing the students’ mathematical creative thinking ability in terms of self-regulated learning: How do we find what we are looking for?
Source: Heliyon. 2024 Jan 20;10(3):e24871. doi: 10.1016/j.heliyon.2024.e24871 (PMC10838748; doi:10.1016/j.heliyon.2024.e24871)
Supplement: Multimedia component 2 [file mmc2.docx]

**SELFREGULATED LEARNING QUESTIONNAIRE SHEET**

1. **Fill in the Personal Identity List Correctly**

Full Name :

Class :

**II. Questionnaire Instructions**

1. Write your full name and class on “Personal Identity”.
2. Read each statement item carefully and thoroughly.
3. Please choose the answer that you think is most appropriate to your situation or opinion, by placing a checklist (√) in the space provided.
4. All answers are acceptable, no explanation is considered wrong and does not affect the assessment.

**Description:**

1. Always (A)
2. Often (O)
3. Sometimes (S)
4. Never (N)

Have a great time doing it ☺

| **No** | **Statement** | **Alternative Answers** | | | |
| --- | --- | --- | --- | --- | --- |
|  |  | **A** | **O** | **S** | **N** |
| 1. | I ask a friend for the answer during a test without thinking about the consequences. |  |  |  |  |
| 2. | I'm lazy to do math assignments because it's hard for me to do. |  |  |  |  |
| 3. | I only fulfill tasks in understanding math lessons. |  |  |  |  |
| 4. | I choose to solve my own math test according to my ability even if it is different from my friends' answers. |  |  |  |  |
| 5. | I have my own target score for every math test I pass. |  |  |  |  |
| 6. | Whenever there are difficulties in learning mathematics, I try to find my own references from books before asking friends. |  |  |  |  |
| 7. | I prepare learning equipment before studying mathematics at school. |  |  |  |  |
| 8. | I just let the assignment given by the teacher go when it is difficult to do. |  |  |  |  |
| 9. | I am confident in my ability to get good results in mathematics |  |  |  |  |
| 10. | I use the library or the internet to study mathematics. |  |  |  |  |
| 11. | I don't care about learning math. |  |  |  |  |
| 12. | I ignore strategies or methods in learning mathematics.. |  |  |  |  |
| 13. | I ignore equipment in learning mathematics. |  |  |  |  |
| 14. | I don't have a learning goal so I choose to play rather than study math. |  |  |  |  |
| 15. | I wait for math materials from friends/teachers rather than looking for them myself. |  |  |  |  |
| 16. | I lack concentration when the teacher asks math questions suddenly |  |  |  |  |
| 17. | I am encouraged to study harder when I get low math test scores. |  |  |  |  |
| 18. | I don't depend on friends when doing math assignments. |  |  |  |  |
| 19. | Examples of math problems from books recommended by the teacher made it easier for me to do math practice problems. |  |  |  |  |
| 20. | I'm confused about where to start in order to study well |  |  |  |  |
| 21 | After the math test, I look back at the answers to the test questions so that I know whether I have answered the test questions correctly or not. |  |  |  |  |
| 22 | I feel unsure about doing math problems if I don't ask others. |  |  |  |  |
| 23 | I determine my own method (reading, note taking, memorizing, practice problems, or others) in understanding the subject matter of mathematics. |  |  |  |  |

**BLUEPRINT OF SELF REGULATED LEARNING**

| **Indicator of Self Regulated Learning** | **No** | **Statements** | |
| --- | --- | --- | --- |
|  |  | **Positive** | **Negative** |
| Have learning initiative | 6 | √ |  |
|  | 12 | √ |  |
| Diagnosing learning needs | 7 | √ |  |
|  | 13 |  | √ |
| Monitor, organize and control learning | 9 | √ |  |
|  | 20 |  | √ |
| Set learning goals or targets | 5 | √ |  |
|  | 14 |  | √ |
| View difficulties as challenges | 2 |  | √ |
| Utilize and search for relevant resources | 10 | √ |  |
|  | 15 |  | √ |
|  | 19 | √ |  |
| Select and apply learning strategies | 3 |  | √ |
|  | 17 |  | √ |
|  | 23 | √ |  |
| Self-determination | 11 |  | √ |
|  | 18 | √ |  |
| self-restraint | 1 |  | √ |
| Make your own decisions | 4 | √ |  |
| Able to solve problems | 8 |  | √ |
| Evaluate the learning process and outcomes | 21 | √ |  |
| Self Efficacy | 16 |  | √ |
|  | 22 |  | √ |
| **Total** | | **11** | **12** |
